# Correlation Between Surgical Ward Stay and High Gentamicin Resistance of *Enterococcus faecalis*: A Retrospective Study

**DOI:** 10.3390/antibiotics15040394

**Published:** 2026-04-13

**Authors:** Luiza Sannikova, Agnieszka Misiewska-Kaczur, Michał Dyaczyński, Bartosz Socha, Georgii Gogichev, Marcin Basiak

**Affiliations:** 1 Department of General, Minimally Invasive and Oncological Surgery, The Silesian Hospital in Cieszyn, 43-400 Cieszyn, Poland; 2 Department of Anesthesiology and Intensive Care, The Silesian Hospital in Cieszyn, 43-400 Cieszyn, Poland; 3 Independent Researcher, 121552 Moskow, Russia; 4 Department of Internal Medicine and Clinical Pharmacology, Medical University of Silesia, 40-752 Katowice, Poland

**Keywords:** antibiotic resistance, HLAR, HLGR, hospital-acquired infections, infection control

## Abstract

**Background:** The increasing antimicrobial resistance of *Enterococcus faecalis*, particularly high-level aminoglycoside resistance, represents a growing challenge in the management of hospital-acquired infections. Surgical wards are considered potential environments for the dissemination of resistant strains due to frequent antibiotic exposure and invasive procedures. **Methods:** The aim of this study was to evaluate the association between hospitalization in a general surgery ward and the detection of *Enterococcus faecalis* isolates with high-level gentamicin resistance (HLGR). A retrospective observational study was conducted using microbiological and clinical data from a single medical center in Poland between 2022 and 2024. Only the first isolate per patient was included in the analysis. HLGR was detected using gentamicin at a screening concentration of 500 µg/mL. Associations between clinical variables and HLGR were assessed using univariate analysis and multivariable logistic regression, including hospitalization in a general surgery ward, age, prior hospitalization, and antibiotic therapy within 90 days. **Results:** HLGR was identified in a substantial proportion of *Enterococcus faecalis* isolates. Hospitalization in a general surgery ward was significantly associated with HLGR detection. In multivariable analysis, surgical ward hospitalization remained independently associated with HLGR after adjustment for other variables. Prior antibiotic exposure demonstrated the strongest association with HLGR. **Conclusions:** Hospitalization in a general surgery ward was associated with an increased likelihood of detecting *Enterococcus faecalis* isolates with high-level gentamicin resistance. These findings support the importance of antimicrobial stewardship and infection control strategies in surgical settings to limit the spread of resistant enterococcal strains.

## 1. Introduction

*Enterococcus faecalis* is a Gram-positive bacterium naturally found in the gastrointestinal tract of humans and animals. Under conditions of weakened immunity or microflora disruption, it can become an opportunistic pathogen, causing various infections, especially in the hospital environment [1]. One of the virulence factors of *E. faecalis* is cytolysin, a pore-forming exotoxin capable of lysing both bacterial and eukaryotic cells in response to quorum signals [2]. *Enterococcus faecalis* causes a wide range of nosocomial infections, including urinary tract infections, endocarditis, and wound infections (including post-burn infections, diabetic ulcer infections, and post-operative wound infections). It is also a common cause of bacteremia [3,4,5]. In recent years, there has been growing concern about the resistance of this microorganism to antibiotics, especially aminoglycosides. The mechanisms of HLAR (high-level aminoglycoside resistance) in *E. faecalis* are associated with genes encoding aminoglycoside-inactivating enzymes, such as gentamicin (the *aph(2″)-Ia* gene encoding a bifunctional enzyme with *2″-phosphotransferase* and *6′-acetyltransferase* activity [6]) and streptomycin (via ribosomal mutations) [7]. The high prevalence of HLAR among clinical isolates of this pathogen significantly complicates the selection of appropriate therapy and increases the risk of clinical failures [8,9,10,11]. Furthermore, enterococci can transfer resistance factors not only among themselves but also to other pathogens. For example, gentamicin resistance can be transferred from enterococci to staphylococci, accelerating the spread of resistance among clinically relevant bacteria [12,13]. Enterococcal infections pose a significant clinical problem due to their ability to adapt to various environmental conditions and form antibiotic-resistant biofilms. *Enterococcus faecalis* and *Enterococcus faecium* are the main etiological agents of nosocomial infections, especially in immunocompromised patients [1]. This, in turn, leads to prolonged hospitalization, increased treatment costs, and increased mortality in patients with enterococcal infections [14,15]. According to a study by Farsi et al. (2023) [14], the increasing resistance of *Enterococcus faecium* and *Enterococcus faecalis*, especially in surgical wards, poses a significant challenge for surgeons in the treatment of postoperative infections. The authors emphasize that most *E. faecium* isolates demonstrate resistance to ampicillin, vancomycin, and aminoglycosides, significantly limiting therapeutic options and worsening patient outcomes [14]. Furthermore, a study by Celik et al. (2024) revealed complex mechanisms of delayed wound healing caused by *Enterococcus faecalis* infection [16]. This pathogen has been shown to modulate the host immune response and disrupt tissue regeneration, which can lead to chronic inflammation and delayed epithelialization. Previous studies in the context of Polish healthcare have examined the susceptibility of *Enterococcus faecalis* to ampicillin and penicillin [17], vancomycin [18], and linezolid resistance [19], it also analyzed the antimicrobial resistance of *Enterococcus spp.* isolated from the urine of hospitalized patients in a Polish clinic during 2016–2021 [20]. These studies have provided a better understanding of the mechanisms of *E. faecalis* resistance to key antibiotics used in clinical practice. A study conducted by Malka et al. (2023), which analyzed the causative agents of chronic wound infections in Poland, revealed that *Enterococcus faecalis* is one of the most frequently isolated microorganisms [21]. Despite growing evidence on the role of antibiotic exposure and prior hospitalization in the development of high-level aminoglycoside resistance, the independent contribution of specific hospital environments, particularly general surgery wards, remains insufficiently characterized. In most studies, surgical departments are analyzed together with other hospital settings or intensive care units, making it difficult to determine their specific role in the epidemiology of resistant enterococci. Moreover, data addressing this issue in Central and Eastern European healthcare systems, including Poland, remain limited, despite known regional variability in antimicrobial resistance patterns. Therefore, the aim of this study was to evaluate whether hospitalization in a general surgery ward is independently associated with the detection of *Enterococcus faecalis* isolates with high-level gentamicin resistance, after adjustment for key clinical risk factors.

## 2. Results

A total of 511 clinical isolates obtained from wound swabs of hospitalized patients were included in the final analysis. These isolates were evaluated for the presence of high-level aminoglycoside resistance (HLAR) and associated clinical factors. The mean age of the patients was 69.0 ± 11.6 years, while the median age was 68 years, indicating that the analyzed population mainly consisted of elderly patients. Age distribution analysis demonstrated relatively symmetric dispersion without extreme outliers. Among all analyzed isolates, HLAR was detected in 194 cases, corresponding to 38.0% of the entire study population. Thus, more than one-third of *Enterococcus* isolates demonstrated high-level resistance to aminoglycosides.

As noted earlier, several potential clinical risk factors were recorded for each patient and subsequently included in the analysis. These variables included:

1. Hospitalization during the previous six months;
2. Antibiotic therapy within 90 days prior to sampling;
3. Hospitalization in the general surgery ward;
4. Age of the patient.

Hospitalization within the preceding six months was recorded in 65 patients (12.7%), whereas 446 patients (87.3%) had no documented hospitalization during that period. Prior antibiotic exposure within 90 days was observed in 108 patients (21.1%), while 403 patients (78.9%) had not received antibiotic therapy during that timeframe. Finally, 99 patients (19.4%) were hospitalized in the general surgery ward, whereas 412 patients (80.6%) were treated in other hospital departments. These baseline characteristics formed the basis for further statistical analysis aimed at identifying factors associated with the occurrence of HLAR.

The primary objective of the study was to evaluate the association between hospitalization in the general surgery ward and the presence of high-level aminoglycoside resistance (HLAR).

To address this objective, patients were divided into two groups according to hospitalization during the previous six months. Among 446 patients without prior hospitalization, HLAR was detected in 150 cases (33.6%), while 296 isolates (66.4%) were HLAR-negative. In contrast, among 65 patients with documented hospitalization within the previous six months, HLAR was detected in 44 cases (67.7%), while 21 isolates (32.3%) were HLAR-negative. Thus, the proportion of HLAR-positive isolates was approximately twice as high in patients with prior hospitalization compared with those without such history. To quantify the strength of this association, an odds ratio (OR) was calculated. The odds of detecting HLAR among patients with previous hospitalization were 4.13 times higher than among patients without hospitalization: OR = 4.13 (95% CI: 2.37–7.21).

The confidence interval did not include the value 1, indicating a statistically significant association between previous hospitalization and HLAR occurrence.

These findings suggest that recent hospitalization represents an important epidemiological factor contributing to the emergence and spread of highly resistant enterococcal strains.

Another important factor analyzed in this study was antibiotic therapy administered within 90 days prior to sample collection. Among 403 patients without prior antibiotic therapy, HLAR was detected in 114 cases (28.3%), whereas 289 isolates (71.7%) remained susceptible to aminoglycosides. In contrast, among 108 patients who had received antibiotics during the previous 90 days, HLAR was detected in 80 cases (74.1%), while only 28 isolates (25.9%) were HLAR-negative.

Thus, the proportion of HLAR-positive isolates in patients previously exposed to antibiotics was more than two and a half times higher than in patients without such exposure. The calculated odds ratio demonstrated a very strong association between prior antibiotic therapy and HLAR occurrence: OR = 7.24 (95% CI: 4.47–11.73).

This result indicates that patients who received antibiotics during the previous 90 days had more than sevenfold increased odds of harboring HLAR-positive enterococcal isolates.

Because the main aim of the study was to assess the role of hospitalization in the general surgery ward, this variable was analyzed separately. Among 412 patients treated in other hospital departments, HLAR was detected in 132 cases (32.0%), whereas 280 isolates (68.0%) were HLAR-negative. In contrast, among 99 patients hospitalized in the general surgery ward, HLAR was detected in 62 cases (62.6%), while 37 isolates (37.4%) were HLAR-negative. Thus, the prevalence of HLAR among patients treated in the general surgery ward was nearly two times higher than among patients treated in other departments. The calculated odds ratio confirmed the statistical significance of this association (OR = 3.55 (95% CI: 2.25–5.61)). These findings suggest that hospitalization in the surgical environment may represent an important risk factor for colonization or infection with highly resistant enterococcal strains.

Several mechanisms may contribute to this observation, including frequent use of broad-spectrum antibiotics, invasive procedures, prolonged hospital stays, and increased exposure to healthcare-associated microorganisms.

Age was also evaluated as a potential predictor of HLAR occurrence. The mean age among patients with HLAR-positive isolates was 68.9 ± 13.3 years, whereas the mean age among patients with HLAR-negative isolates was 69.1 ± 10.5 years.

Statistical comparison did not reveal a significant difference between the groups. This finding indicates that patient age was not associated with HLAR occurrence in the analyzed population. Therefore, age was not considered a major determinant of high-level aminoglycoside resistance in this cohort.

To determine whether hospitalization remained independently associated with HLAR after controlling for other potential risk factors, a multivariate logistic regression model was constructed.

The following variables were included in the model:

1. Hospitalization within the previous six months;
2. Antibiotic therapy within 90 days;
3. Hospitalization in the general surgery ward;
4. Age.

The results of the multivariate analysis demonstrated that several factors remained statistically significant predictors of HLAR.

The strongest independent predictor was prior antibiotic therapy: adjusted OR (aOR) = 6.29 (95% CI: 3.80–10.42; *p* < 0.001).

Patients with previous antibiotic exposure had more than sixfold increased odds of harboring HLAR-positive isolates.

Hospitalization within the previous six months also remained an independent predictor: aOR = 3.59 (95% CI: 1.96–6.58; *p* < 0.001).

This finding indicates that the association between hospitalization and HLAR cannot be explained solely by antibiotic exposure.

Hospitalization in the general surgery ward was likewise independently associated with HLAR: aOR = 2.23 (95% CI: 1.34–3.71; *p* = 0.002).

This suggests that the surgical hospital environment itself may contribute to the dissemination of resistant enterococcal strains.

Age did not demonstrate a statistically significant effect: aOR = 0.92 per 10-year increase (95% CI: 0.77–1.09; *p* = 0.32). Thus, age was not retained as a meaningful predictor in the final interpretation of the results.

Summary of key findings:

The present analysis demonstrated several findings regarding the epidemiology of high-level aminoglycoside resistance among enterococcal isolates.

First, HLAR was detected in 38% of all isolates, indicating a relatively high prevalence of resistance in the studied population.

Second, previous hospitalization within six months was strongly associated with HLAR, increasing the odds of resistance by more than four times in the univariate analysis and nearly four times after adjustment for confounding factors.

Third, prior antibiotic therapy emerged as the most important predictor of HLAR, increasing the odds of resistance more than sixfold in the multivariate model.

Fourth, hospitalization in the general surgery ward was also independently associated with HLAR, suggesting that surgical settings may play a role in the transmission or selection of resistant enterococci.

Finally, patient age was not associated with HLAR occurrence, indicating that resistance patterns were primarily determined by healthcare-related factors rather than demographic characteristics.

Together, these findings highlight the important role of healthcare exposure and antibiotic pressure in the development and dissemination of high-level aminoglycoside resistance among enterococci.

## 3. Discussion

The key novel finding of this study is that hospitalization in a general surgery ward remained independently associated with the detection of high-level gentamicin-resistant *Enterococcus faecalis* isolates after adjustment for prior antibiotic exposure and recent hospitalization. This may suggest that the surgical ward should not be interpreted solely as a proxy for antibiotic pressure, but rather as a distinct clinical and ecological environment potentially contributing to the persistence and spread of resistant enterococcal strains. Several mechanisms may plausibly explain this association, including antibiotic selection pressure related to frequent use of broad-spectrum agents, as well as environmental exposure and cross-transmission within the hospital setting [12,22,23]. However, due to the retrospective design and the lack of precise temporal data linking ward exposure to microbiological sampling, these mechanisms remain hypothetical. In the multivariable analysis, prior antibiotic therapy showed the strongest association with HLGR, while hospitalization in a general surgery ward demonstrated a more moderate but still independent association. This may indicate that antibiotic exposure plays a major role in the selection of resistant strains, whereas surgical ward hospitalization may represent an additional contributing factor.

High resistance of *Enterococcus faecalis* observed in the present study is consistent with findings reported in numerous studies conducted both in Poland and internationally. Previous research has demonstrated that enterococci represent an important group of opportunistic pathogens in hospital settings and are increasingly associated with multidrug resistance. For example, a study conducted in Poland by Gawryszewska et al., analyzing invasive isolates of *Enterococcus faecalis* and *Enterococcus faecium* from multiple medical centers, demonstrated a high prevalence of aminoglycoside resistance and confirmed the clinical significance of these pathogens [24].

Similarly, a multicenter study by Chmielarczyk et al. reported a high prevalence of resistant Gram-positive pathogens in hospitalized patients, including enterococci demonstrating the HLAR mechanism, particularly in intensive care and surgical wards [25]. Other studies have also shown that highly resistant enterococcal strains are most frequently isolated from patients hospitalized in surgical units, intensive care units, and oncology departments, where exposure to invasive procedures and broad-spectrum antibiotic therapy is common [26,27].

At the European level, surveillance data from the European Centre for Disease Prevention and Control indicate that high-level gentamicin resistance among *Enterococcus faecalis* isolates remains a persistent problem across many EU and EEA countries, with an average prevalence of approximately 29%, although significant regional differences have been observed [22,28].

Similar findings have been reported in studies conducted in Asia and the Middle East, which also demonstrate high levels of aminoglycoside resistance among enterococcal isolates. Genetic analyses indicate that this resistance is frequently associated with aminoglycoside-modifying enzyme genes such as *aac(6′)-Ie-aph(2″)-Ia* and *aph(3′)-IIIa*, which play a key role in the development of high-level resistance to aminoglycosides [29,30,31,32].

Enterococci possess numerous mechanisms that facilitate their survival in hospital environments, including intrinsic resistance to many antimicrobial agents, the ability to acquire additional resistance determinants, and the capacity to form biofilms. These characteristics enable them to persist on medical devices and surgical implants and contribute to the development of difficult-to-treat infections [33,34].

Although antibiotic exposure is considered an important factor contributing to the emergence of aminoglycoside-resistant enterococci, precise thresholds regarding the duration or dosage of antibiotic therapy leading to the development of HLAR have not been clearly established in the current literature. Several studies suggest that prolonged exposure to aminoglycosides and other broad-spectrum antibiotics may promote the selection and spread of resistant Enterococcus strains in hospital environments [23]. Surveillance data from the European Centre for Disease Prevention and Control also confirm that high-level gentamicin resistance among *Enterococcus faecalis* remains a persistent problem across many EU and EEA countries [22,28]. These findings highlight the importance of careful antimicrobial stewardship and monitoring of antibiotic use in hospital settings.

It should be noted that the detection of *Enterococcus faecalis* in wound cultures does not always indicate an active infection. As part of the normal human microbiota, enterococci may colonize various anatomical sites, including the gastrointestinal tract and skin, and may also be present in wounds without causing clinically significant infection. Therefore, the interpretation of microbiological findings should always be performed in the context of the patient’s clinical condition and other diagnostic parameters [1,35]. Distinguishing colonization from true infection is essential for appropriate treatment decisions. Clinical signs such as local inflammation, purulent discharge, delayed wound healing, and systemic inflammatory markers may support the diagnosis of infection, whereas the detection of low bacterial counts without clinical symptoms may indicate colonization rather than active disease [3,36]. Another important factor that may influence microbiological results is contamination during sample collection. Improper sampling techniques or superficial swabs may lead to the detection of colonizing microorganisms originating from surrounding skin or the environment. For this reason, proper wound preparation and deep tissue sampling are recommended to improve diagnostic accuracy and reduce the risk of contamination [16,37,38]. The presence of mixed microbial flora may also complicate the interpretation of microbiological findings. When several microorganisms are detected, and *Enterococcus faecalis* is not the dominant species, its clinical significance may be limited and may reflect colonization rather than infection [1,16]. In the context of the present study, these factors should be considered when interpreting microbiological results, particularly because sampling practices may vary between hospital wards. While wound samples in the surgical ward were collected according to standardized procedures consistent with clinical recommendations, it was not possible to verify whether identical sampling protocols were applied in other departments due to the retrospective nature of the study. Several limitations of this study should be acknowledged. First, the single-center design may limit the generalizability of the findings, as local antimicrobial prescribing practices, patient characteristics, and infection control measures may differ from those in other institutions. Second, the retrospective nature of the analysis may introduce information bias and limit the ability to control for all potential confounding factors. In particular, detailed data on duration of hospitalization, invasive procedures, and specific antibiotic regimens were not consistently available and therefore could not be included in the analysis. This may have resulted in residual confounding and may influence the observed associations. Importantly, due to the lack of precise temporal data linking ward exposure to microbiological sampling, it cannot be determined whether HLGR was present prior to hospitalization or developed during the hospital stay. This limits the interpretation of the directionality of the observed association. Therefore, the findings should be interpreted as associations rather than evidence of causal relationships. Further multicenter studies, including a broader range of clinical variables, may provide more comprehensive insight into the epidemiology of HLGR in *Enterococcus faecalis*.

## 4. Materials and Methods

Study design and data collection:

A retrospective observational study was conducted to evaluate the association between hospitalization in a general surgery ward and the presence of high-level aminoglycoside resistance (HLAR) in *Enterococcus faecalis* isolates. The study was based on microbiological and clinical data obtained from hospital records between January 2022 and December 2024.

Microbiological data were extracted from the hospital laboratory information system, while selected clinical variables were retrieved from electronic medical records. Only the first isolate obtained from each patient during the study period was included in the analysis to avoid duplication.

The primary objective of the study was to determine whether hospitalization in a general surgery ward is associated with an increased likelihood of HLAR in *Enterococcus faecalis* isolates.

Study population:

Patients were divided into two groups according to the presence of high-level aminoglycoside resistance:

- HLAR-positive group—patients with *Enterococcus faecalis* isolates demonstrating high-level aminoglycoside resistance.
- HLAR-negative group—patients with isolates susceptible to high concentrations of aminoglycosides.

The primary exposure variable was hospitalization in a general surgery ward, while patients hospitalized in other departments served as the comparison group.

Additional clinical variables analyzed as potential risk factors included:

- Patient age;
- Hospitalization within the previous six months;
- Antibiotic therapy within 90 days prior to microbiological sampling.

Inclusion and exclusion criteria:

Patients were included in the study if they met the following criteria:

- Age ≥ 18 years;
- Availability of microbiological identification of *Enterococcus faecalis* from clinical samples;
- Availability of antimicrobial susceptibility testing results, including screening for high-level gentamicin resistance.

Exclusion criteria:

Patients were excluded from the study if:

- Microbiological or clinical data required for analysis were incomplete;
- Antimicrobial susceptibility testing results were unavailable;
- Repeated isolates from the same patient were detected during the same hospitalization episode. In such cases, only the first isolate per patient was included in the analysis.

Microbiological methods:

Identification of *Enterococcus faecalis* isolates was performed in the hospital microbiology laboratory using standard diagnostic procedures. Bacterial isolates were cultured on selective media and identified using conventional biochemical methods in accordance with routine laboratory protocols.

Antimicrobial susceptibility testing was performed using standard microbiological methods recommended for clinical laboratories.

High-level aminoglycoside resistance (HLAR) was determined by screening for high-level gentamicin resistance (HLGR) using gentamicin at a concentration of 500 µg/mL. During the study period, routine testing for high-level streptomycin resistance was not performed in the microbiology laboratory. Therefore, in this study, the term HLAR refers specifically to high-level gentamicin resistance.

Based on susceptibility testing results, isolates were classified as either:

- HLAR-positive;
- HLAR-negative.

Ethical considerations:

The study was conducted in accordance with the principles of the Declaration of Helsinki. All collected data were anonymized prior to analysis to ensure patient confidentiality. No personally identifiable information was included in the dataset used for the study.

Because the study used retrospective anonymized data obtained from routine clinical practice, no additional intervention involving patients was performed.

Statistical analysis:

Statistical analysis was performed using Python software with the pandas, scipy, and statsmodels libraries.

Descriptive statistics were used to summarize baseline characteristics of the study population. Continuous variables were presented as mean ± standard deviation, while categorical variables were expressed as numbers and percentages.

Associations between categorical variables were evaluated using contingency tables. The strength of associations between potential risk factors and the presence of HLAR was assessed by calculating odds ratios (ORs) with 95% confidence intervals (95% CI).

To identify independent predictors of HLAR, a multivariate logistic regression model was constructed, including the following variables:

- Hospitalization within the previous six months;
- Antibiotic therapy within 90 days;
- Hospitalization in a general surgery ward;
- Patient age.

Statistical significance was defined as a *p*-value < 0.05.

## 5. Conclusions

Hospitalization in a general surgery ward was independently associated with the detection of high-level gentamicin-resistant *Enterococcus faecalis* isolates, although the strongest association was observed for prior antibiotic exposure. These findings may suggest that, in addition to antibiotic selection pressure, the surgical ward environment could represent an additional factor potentially contributing to the persistence and spread of resistant strains.

From a clinical perspective, these findings may support consideration of the following strategies in surgical settings:

- Routine reassessment and early de-escalation of antibiotic therapy, particularly in patients with recent antibiotic exposure;
- Reinforcement of infection prevention measures, including hand hygiene, environmental cleaning, and aseptic technique;
- Consideration of contact precautions or patient cohorting in accordance with local protocols;
- Targeted microbiological surveillance in patients with prior hospitalization or repeated healthcare exposure;
- Closer collaboration with antimicrobial stewardship and infection control teams; consideration of developing and implementing local, hospital-based antibiotic guidelines tailored to resistance patterns.

Further multicenter prospective studies may help to better understand the mechanisms underlying these associations and to support the development of optimized prevention strategies.

## Data Availability

The data presented in this study are available on request from the corresponding author (the data are not publicly available due to privacy or ethical restrictions).

## References

[1] Said M.S., Tirthani E., Lesho E. (2024). Enterococcus Infections. StatPearls.

[2] Van Tyne D., Martin M.J., Gilmore M.S. (2013). Structure, function, and biology of the *Enterococcus faecalis* cytolysin. Toxins.

[3] Chong K.K.L., Tay W.H., Janela B., Yong A.M.H., Liew T.H., Madden L., Keogh D., Barkham T.M.S., Ginhoux F., Becker D.L. (2017). *Enterococcus faecalis* Modulates Immune Activation and Slows Healing During Wound Infection. J. Infect. Dis..

[4] Kontula K.S.K., Skogberg K., Ollgren J., Järvinen A., Lyytikäinen O. (2021). Population-Based Study of Bloodstream Infection Incidence and Mortality Rates, Finland, 2004–2018. Emerg. Infect. Dis..

[5] Rosselli Del Turco E., Bartoletti M., Dahl A., Cervera C., Pericàs J.M. (2021). How do I manage a patient with enterococcal bacteraemia?. Clin. Microbiol. Infect..

[6] Mederski-Samoraj B.D., Murray B.E. (1983). High-level resistance to gentamicin in clinical isolates of enterococci. J. Infect. Dis..

[7] Eliopoulos G.M., Farber B.F., Murray B.E., Wennersten C., Moellering R.C. (1984). Ribosomal resistance of clinical enterococcal to streptomycin isolates. Antimicrob. Agents Chemother..

[8] Amini F., Krimpour H.A., Ghaderi M., Vaziri S., Ferdowsi S., Azizi M., Amini S. (2018). Prevalence of Aminoglycoside Resistance Genes in *Enterococcus* Strains in Kermanshah, Iran. Iran. J. Med. Sci..

[9] Coll F., Gouliouris T., Blane B., Yeats C.A., Raven K.E., Ludden C., Khokhar F.A., Wilson H.J., Roberts L.W., Harrison E.M. (2024). Antibiotic resistance determination using *Enterococcus faecium* whole-genome sequences: A diagnostic accuracy study using genotypic and phenotypic data. Lancet Microbe.

[10] Harada S., Shibue Y., Aoki K., Ishii Y., Tateda K. (2020). Prevalence of High-Level Aminoglycoside Resistance and Genes Encoding Aminoglycoside-Modifying Enzymes in *Enterococcus faecalis* and *Enterococcus faecium* Isolated in a University Hospital in Tokyo. Jpn. J. Infect. Dis..

[11] Osuka H., Nakajima J., Oishi T., Funayama Y., Ebihara T., Ishikawa H., Saito K., Koganemaru H., Hitomi S. (2016). High-level aminoglycoside resistance in *Enterococcus faecalis* and *Enterococcus faecium* causing invasive infection: Twelve-year surveillance in the Minami Ibaraki Area. J. Infect. Chemother..

[12] Hegstad K., Mikalsen T., Coque T.M., Werner G., Sundsfjord A. (2010). Mobile genetic elements and their contribution to the emergence of antimicrobial resistant *Enterococcus faecalis* and *Enterococcus faecium*. Clin. Microbiol. Infect..

[13] Noble W.C., Rahman M., Karadec T., Schwarz S. (1996). Gentamicin resistance gene transfer from *Enterococcus faecalis* and *E. faecium* to *Staphylococcus aureus*, *S. intermedius* and *S. hyicus*. Vet. Microbiol..

[14] Farsi S., Salama I., Escalante-Alderete E., Cervantes J. (2023). Multidrug-Resistant Enterococcal Infection in Surgical Patients, What Surgeons Need to Know. Microorganisms.

[15] Puchter L., Chaberny I.F., Schwab F., Vonberg R.P., Bange F.C., Ebadi E. (2018). Economic burden of nosocomial infections caused by vancomycin-resistant enterococci. Antimicrob. Resist. Infect. Control.

[16] Celik C., Lee S.T.T., Tanoto F.R., Veleba M., Kline K., Thibault G. (2024). Decoding the complexity of delayed wound healing following *Enterococcus faecalis* infection. eLife.

[17] Gawryszewska I., Żabicka D., Hryniewicz W., Sadowy E. (2021). Penicillin-resistant, ampicillin-susceptible *Enterococcus faecalis* in Polish hospitals. Microb. Drug Resist..

[18] Sadowy E., Sieńko A., Gawryszewska I., Bojarska A., Malinowska K., Hryniewicz W. (2013). High abundance and diversity of antimicrobial resistance determinants among early vancomycin-resistant *Enterococcus faecium* in Poland. Eur. J. Clin. Microbiol. Infect. Dis..

[19] Krawczyk B., Wysocka M., Kotłowski R., Bronk M., Michalik M., Samet A. (2020). Linezolid-resistant *Enterococcus faecium* strains isolated from one hospital in Poland–commensals or hospital-adapted pathogens?. PLoS ONE.

[20] Kraszewska Z., Skowron K., Kwiecińska-Piróg J., Grudlewska-Buda K., Przekwas J., Wiktorczyk-Kapischke N., Wałecka-Zacharska E., Gospodarek-Komkowska E. (2022). Antibiotic Resistance of *Enterococcus* spp. Isolated from the Urine of Patients Hospitalized in the University Hospital in North-Central Poland, 2016–2021. Antibiotics.

[21] Malka M., Krakowiecki A., Chojak M., Pławski M., Wądołek M., Wołowicz A., Dyczewska A., Paź A., Pawlik K., Grzela T. (2023). The Pathogen Isolates in Chronic Wound Infections in Poland. Pol. J. Microbiol..

[22] (2023). Antimicrobial Resistance Surveillance in Europe 2023–2021 Data.

[23] Arias C.A., Murray B.E. (2012). The rise of the Enterococcus: Beyond vancomycin resistance. Nature reviews. Microbiology.

[24] Gawryszewska I., Żabicka D., Bojarska K., Malinowska K., Hryniewicz W., Sadowy E. (2016). Invasive enterococcal infections in Poland: The current epidemiological situation. Eur. J. Clin. Microbiol. Infect. Dis..

[25] Chmielarczyk A., Pomorska-Wesołowska M., Romaniszyn D., Wójkowska-Mach J. (2021). Healthcare-Associated Laboratory-Confirmed Bloodstream Infections-Species Diversity and Resistance Mechanisms, a Four-Year Retrospective Laboratory-Based Study in the South of Poland. Int. J. Environ. Res. Public Health.

[26] Kozuszko S., Białucha A., Bogiel T., Gospodarek E. (2011). Resistance to high concentrations of aminoglycosides among *Enterococcus faecalis* and *Enterococcus faecium* strains isolated from clinical material. Exp. Med. Microbiol..

[27] Vigani A.G., Oliveira A.M., Bratfich O.J., Stucchi R.S., Moretti M.L. (2008). Clinical, epidemiological, and microbiological characteristics of bacteremia caused by high-level gentamicin-resistant *Enterococcus faecalis*. Braz. J. Med. Biol. Res. = Rev. Bras. Pesqui. Medicas Biol..

[28] European Centre for Disease Prevention and Control (ECDC) (2024). Antimicrobial Resistance in the EU/EEA (EARS-Net Annual Epidemiological Report 2024).

[29] Diab M., Salem D., El-Shenawy A., El-Far A., Abdelghany A., Awad A.R., El Defrawy I., Shemis M. (2019). Detection of high level aminoglycoside resistance genes among clinical isolates of Enterococcus species. Egypt. J. Med. Hum. Genet..

[30] Khodabandeh M., Mohammadi M., Abdolsalehi M.R., Hasannejad-Bibalan M., Gholami M., Alvandimanesh A., Pournajaf A., Rajabnia R. (2020). High-Level Aminoglycoside Resistance in Enterococcus faecalis and *Enterococcus faecium*; as a Serious Threat in Hospitals. Infect. Disord. Drug Targets.

[31] Moussa A.A., Md Nordin A.F., Hamat R.A., Jasni A.S. (2019). High level aminoglycoside resistance and distribution of the resistance genes in *Enterococcus faecalis* and *Enterococcus faecium* from teaching hospital in Malaysia. Infect. Drug Resist..

[32] Padmasini E., Padmaraj R., Ramesh S.S. (2014). High level aminoglycoside resistance and distribution of aminoglycoside resistant genes among clinical isolates of Enterococcus species in Chennai, India. Sci. World J..

[33] Esmail M.A.M., Abdulghany H.M., Khairy R.M. (2019). Prevalence of Multidrug-Resistant *Enterococcus faecalis* in Hospital-Acquired Surgical Wound Infections and Bacteremia: Concomitant Analysis of Antimicrobial Resistance Genes. Infect. Dis..

[34] Silva V., Freitas C., Ribeiro J., Igrejas G., Poeta P. (2025). Comparative analysis of antibiotic resistance and biofilm formation in Enterococcus spp. across One Health domains. FEMS Microbes.

[35] Zirakzadeh A., Patel R. (2006). Vancomycin-resistant enterococci: Colonization, infection, detection, and treatment. Mayo Clin. Proc..

[36] Goh H.M., Yong M.H., Chong K.K., Kline K.A. (2017). Model systems for the study of Enterococcal colonization and infection. Virulence.

[37] Berenguer-Pérez M., Manzanaro-García N., González-de la Torre H., Durán-Sáenz I., Hernández Martínez-Esparza E., Diaz Herrera M.Á., González Suárez B., Verdú-Soriano J. (2024). Systematic review and meta-analysis of diagnostic test accuracy in chronic wound’s microbiology. Int. Wound J..

[38] Rembe J.D., Garabet W., Lackmann J.W., Alizadehrahrouei S., Augustin M., Dissemond J., Ibing W., Köhrer K., Pfeffer K., Rommerskirchen A. (2024). Assessment and Monitoring of the Wound Micro-Environment in Chronic Wounds Using Standardized Wound Swabbing for Individualized Diagnostics and Targeted Interventions. Biomedicines.

